# Mixed *Bacillus subtilis* and *Lactiplantibacillus plantarum*-fermented feed improves gut microbiota and immunity of Bamei piglet

**DOI:** 10.3389/fmicb.2024.1442373

**Published:** 2024-08-29

**Authors:** Jun Chen, Liyu Mou, Lei Wang, Guofang Wu, Ximei Dai, Qiufang Chen, Jianbo Zhang, Xuan Luo, Fafang Xu, Miao Zhang, Yaoke Duan, Huili Pang, Yanping Wang, Yimin Cai, Zhongfang Tan

**Affiliations:** ^1^Henan Key Laboratory of Ion-Beam Green Agriculture Bioengineering, School of Agricultural Sciences, Zhengzhou University, Zhengzhou, China; ^2^Plateau Livestock Genetic Resources Protection and Innovative Utilization Key Laboratory of Qinghai Province, Key Laboratory of Animal Genetics and Breeding on Tibetan Plateau, Ministry of Agriculture and Rural Affairs, Qinghai Academy of Animal Science and Veterinary Medicine, Qinghai University, Xining, China; ^3^Laboratory of Zhongyuan Light, School of Physics, Zhengzhou University, Zhengzhou, China; ^4^Bamei Pig Original Breeding Base of Huzhu County, Huzhou, China

**Keywords:** probiotics, piglet, fermented feed, intestinal microbiota, immunity

## Abstract

Antibiotics are widely used in the breeding production of Bamei pigs, affecting the quality and safety of pork and causing enormous harm to human health, the environment, and public health. The use of probiotic fermented feed to replace antibiotic feed is one of the solutions, which has the potential to improve the intestinal microbiota, promote animal growth, and enhance immunity. The purpose of this study was to evaluate the effect of fermented feed with *Lactiplantibacillus* (L.) *plantarum* QP28-1a or *Bacillus* (B.) *subtilis* QB8a on feed, growth performance, gut microbiota, and immunity of weaned piglets. A total of 60 freshly weaned piglets from the Tibetan Plateau were randomly divided into five groups and fed basal feed, *L. plantarum* fermented feed, *B. subtilis* fermented feed, mixed fermented feed, and antibiotic fermented feed for 60 days, respectively. The results showed fermented feed supplemented with *L. plantarum* QP28-1a or *B. subtilis* QB8a significantly lowered the pH of the feed (*P* < 0.05), produced lactic acid and acetic acid, inhibited the growth of harmful bacteria in the feed, and reduced the feed conversion rate in the group fed mixed fermented feed (*P* < 0.05). The fermented feed increased the α-diversity and prominently altered the β-diversity of the intestinal microbiota, increasing the relative abundance of beneficial bacteria such as *Lactobacillus* and *Turicibacter* and decreasing the relative abundance of conditional pathogens such as *Streptococcus* and *Clostridium*, improving the intestinal microbiota of the Bamei piglets. Notably, the mixed fermented feed improved the immunity of Bamei piglets by modulating the production of pro-inflammatory cytokines, anti-inflammatory cytokines, and inflammatory-related signaling pathways. Spearman’s correlation analysis revealed that the increased expression of immune-related cytokines may be associated with a significant enrichment of *Lactobacillus*, *Prevotellaceae*, *Erysipelotrichaceae*, and *Ruminococcaceae* in the gut. In conclusion, the probiotic fermented feed maintained an acidic environment conducive to suppressing pathogens, reduced the feed conversion ratio, optimized the intestinal microbiota, improved immunity, and alleviated intestinal inflammation that may be caused by weaning, demonstrating the excellent application prospects of *L. plantarum* QP28-1a and *B. subtilis* QB8a fermented feed in the feeding of Bamei piglets.

## Graphical Abstract

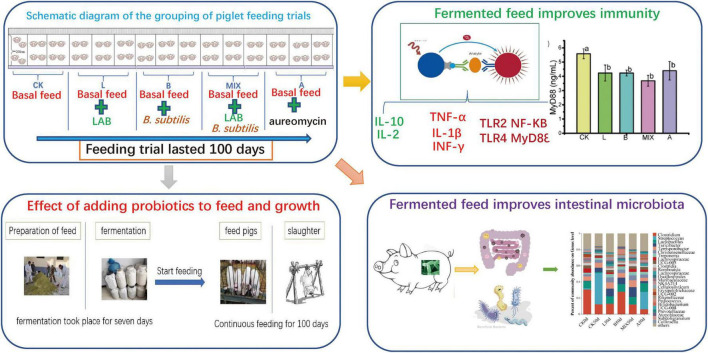

## 1 Introduction

The application of rapid pig fattening technology, the use of antibiotics and various chemical reagents in feed have created lucrative benefits but also brought many problems, such as drug residues, antibiotic resistance, and reduced pork quality (Thacker, 2013; Du et al., 2017). As a result, many countries and regions around the world have proposed restrictions on the use of antibiotics (Baurhoo et al., 2007), which means that the feed industry has entered the era of “no antibiotics” and more researchers are seeking to develop various alternatives to antibiotics and green and harmless feed additives to eliminate or mitigate a series of impacts brought by the ban on antibiotics.

The piglet is the most critical part of the pig breeding process, and its proper feeding directly affects the whole growth period of the pig and determines the production efficiency. However, piglets at the weaning stage are challenged by the weaning stress syndrome. Weaning stress causes intestinal disorders, low feed intake, low immunity, diarrhea, and a significant reduction in growth performance or even mass mortality, which seriously affects the healthy growth of piglets (Shen et al., 2009). As a kind of ecological and healthy feed, fermented feed can not only improve the absorption level of feed nutrients, degrade the anti-nutritional factors and toxic and harmful substances, but also promote animal growth, maintain the micro-ecological balance in the animal’s intestinal tract and enhance the immunity of the body (Canibe and Jensen, 2003; Hojberg et al., 2003). Probiotics and their metabolites are ideal alternatives that have a positive effect on preventing diseases and improving the performance of livestock and poultry (Buesing and Zeyner, 2015; Barathikannan et al., 2019; Yaqoob et al., 2022).

Lactic acid bacteria (LAB), the most widely used probiotic, has the advantage of regulating the intestinal micro-ecological environment of animals, inhibiting the reproduction of harmful bacteria, and lowering the pH value of the digestive tract, which has health benefits in poultry and livestock farming (Yu et al., 2008; Le Bon et al., 2010; Gebert et al., 2011). The advantages of LAB fermented feed include the following aspects: (a) Improve the micro-ecological environment of the gastrointestinal tract, reduce the incidence of the gastrointestinal diseases. LAB can change the internal environment of the gastrointestinal tract, inhibit the reproduction of harmful bacteria, adjust the balance of the gastrointestinal microbiota through fermentation and acid production, and can also be planted on the surface of the intestinal mucosa to become the main part of physiological barriers, so as to achieve the effects of restoring host resistance, repairing the intestinal bacterial barriers, and treating intestinal diseases (Humer et al., 2014). (b) Improve the nutritional level and palatability of feed. LAB can not only directly provide the host with a variety of available essential amino acids and a variety of vitamins and digestive enzymes, but also improve the digestibility and absorption of minerals, thus enhancing the nutritional metabolism of animals (Li et al., 2020), LAB fermented feed can produce a unique fermented sour flavor and improve the palatability of feed for animals (Koo et al., 2018). (c) Enhances the immunity of animals and improves their health (Delcenserie et al., 2008; Li et al., 2024). On the one hand, the increase of beneficial bacteria can inhibit and crowd out harmful bacteria, so that the gastrointestinal tract gradually establishes the advantage of beneficial microbiota. In addition, LAB will produce a series of active substances to directly inhibit the harmful bacteria, reducing the occurrence of disease (van Winsen et al., 2001a). On the other hand, LAB stimulates non-specific and specific immune responses in animals, inducing the production of interferon, promoting cell division, antibody production, and cellular immunity, and improving the body’s ability to resist diseases (Chuang et al., 2007). *B. subtilis* is also a common strain in fermented feed additives. In the process of feed fermentation, *B. subtilis* can produce some metabolites such as protease and other hydrolytic enzymes, which will hydrolyze the cellulose, protein and other macromolecules in the original roughage into glucose, peptide, etc., thus improving the nutrition level of the original feed, and also inhibiting the reproduction of the harmful bacterial strains in the intestinal tract, so as to improve the resistance of the animals (Santoso et al., 1995; Dai et al., 2017). Although there were many previous studies on fermented feed, few people comprehensively investigated and analyzed the effects of probiotics fermented feed from multiple perspectives, including feed, animal growth performance, intestinal microbiota, and immunity.

Considering the positive effects of probiotic fermented feed on animal growth, intestinal health, and immunity, we hypothesize that probiotic fermented feed can improve the intestinal microorganism environment, promote growth, and improve the immunity of Bamei piglets. The present study was therefore conducted to explore the effects of *Lactiplantibacillus* (L.) *plantarum* and *Bacillus* (B.) *subtilis* fermented feed on fermented feed, growth performance, intestinal microbiota, and immunity. In this study, we used *L. plantarum* QP28-1a and *B. subtilis* QB8a, which were previously screened from the intestinal tract of Bamei piglets and have broad-spectrum antibacterial activity and produce bacteriocins (Chen et al., 2022), to make fermented feed for Bamei piglets from the high altitude and cold Tibetan Plateau region. As the only local fine pig breed on the Tibetan Plateau, it has the advantages of excellent meat quality and roughage tolerance, and has an irreplaceable role for other breeds in the Tibetan Plateau region (Zhang et al., 2018). The observed results will be theoretically important for understanding the beneficial functions of the fermented feed with complex probiotics and the performance of replacing antibiotics, as well as providing a theoretical basis for the green and efficient breeding of the Bamei piglets.

## 2 Materials and methods

### 2.1 Stains and feed

The strains *L. plantarum* QP28-1a and *B. subtilis* QB8a for fermented feed were isolated, screened, and identified by the Henan Key Laboratory of Ion-Beam Bioengineering from Zhengzhou University in China. It was known from previous studies that *L. plantarum* QP28-1a has broad-spectrum bacterial inhibitory activity and bacteriocin-producing ability (Chen et al., 2022), and *B. subtilis* QB8a has high protease and cellulase activity. The activation and preparation of the strains were carried out according to the method of Pang et al. (2011). The basal feed was prepared with reference to the Chinese NY/T 65-2004 Standard for Pig Feeding and the results of previous trials (Wu et al., 2022). The dry base feed composition contains 53.72% corn, 14.73% wheat bran, 11.10% soybean meal, and other materials, as presented in Table 1. The *L. plantarum* or *B. subtilis* fermented feed was made by adding 50 kg of water per 100 kg of the base feed with *L. plantarum* or *B. subtilis* at an addition of 1 × 10^6^ CFU/g, respectively, mixing well, sealing in a bucket, and fermenting at room temperature (15–25°C) for 7 days. *L. plantarum* and *B. subtilis* mixed fermenting feeds were made by adding the above two bacteria to the feed at a ratio of 1:1, both at an addition of 1 × 10^6^ CFU/g, and fermenting at room temperature (15–25°C) for 7 days. 50 mg/kg of aureomycin (Huazhong Zhengda Co., Ltd.) was added to the base feed to make antibiotic feed. We started to feed the weaned piglets after 7 days of fermentation and continued to feed them for 100 days until slaughter.

**TABLE 1:** Basal feed ingredients and nutrient content.

Material (%)		Nutrition level^2^	
Corn	53.72	Metabolizable energy (Kcal/kg)	3073
Wheat bran	14.73	Crude protein (%)	13.95
Soybean meal	11.10	Ca (%)	0.58
Alfalfa silage	10.00	Available phosphorus (%)	0.22
Rapeseed meal	4.15	Total phosphorus (%)	0.45
Soybean oil	2.43	Lysine (%)	0.73
Rape stalk	0.80	Methionine (%)	0.23
Stone powder	1.13		
Dicalcium phosphate	0.29		
Salt	0.50		
Lysine	0.15		
Premix^1^	1		

^1^Premix provided per kilogram of diet: VA 16000 IU, VD 33000 IU, VE 35 mg, VK 33 mg, VB 12.5 mg, VB 26 mg, VB 63 mg, VB 120.25 mg, Nicotinic acid 25 mg, Pantothenic acid 15 mg, Biotin 0.15 mg, Cu 150 mg, Fe 80 mg, Zn 80 mg, Mn 10 mg, Se 0.2 mg.

^2^Nutrient levels were measured by near-infrared reflectance spectroscopy.

### 2.2 Animals and study design

A total of 60 healthy, freshly weaned, 30-day-old Bamei ternary hybrid pig (Duroc × Landrace × Bamei), half male and half female, of similar genetic background were selected. Prior to the study, all weaned piglets were fed a week of dairy pig creep feed (Beibei Milk from Beijing Dabeinong Technology Group Co., Ltd., China) to adapt to weaning stress. All 60 piglets were randomly assigned to five treatment groups with three replicates in each treatment group, fed in 15 pigpens with 4 piglets each. The experimental groups were as follows: (1) control Group, fed a basal diet, noted as Group CK; (2) Group L was fed *L. plantarum* QP28-1a fermented feed; (3) Group B was fed *B. subtilis* fermented feed; (4) Group MIX was fed a mixed fermented feed; (5) Group A was fed antibiotic feed. The piglets were numbered, vaccinated, and dewormed. During the trial period, the piglets were allowed to feed and drink freely, and were fed between 8:00 and 19:00 daily, four times a day, with each feeding limited to a little leftover food. The housing was regularly disinfected and cleaned daily to keep it clean, dry, and naturally ventilated. The amount of food taken and leftovers were accurately recorded daily and weighed at the beginning and end of the trial.

### 2.3 Growth performance and sampling

Body weight and average daily feed intake (ADFI) were recorded weekly. Average daily gain (ADG), ADFI, and feed conversion ratio (FCR) were calculated for each group throughout the trial period according to the initial weight (day 0), final weight (day 60), and feed intake. The FCR is calculated as the ratio of feed consumed (in kg) to weight gain (in kg) during the production period. Five different treatment groups were fed different diets for 60 days, and the characteristics of the feed, growth performance, intestinal microbiota changes, and immunity were measured and analyzed at the pre-, mid-, and end of the experiment. On days 0, 10, 20, 30, 40, and 50, nine fresh fecal samples were collected randomly from each treatment group (3 fecal samples from each pigpen) and stored at −80°C in sterilized sampling tubes for subsequent analysis of microbial counts, microbial diversity and variability, immunity, etc.

### 2.4 Analysis of fermentation products and microbiota

In order to investigate the effect of the addition of *L. plantarum* or *B. subtilis* and the addition of antibiotics on the fermentation products, the pH, the organic acid content, and the microbial population of the feed were counted and analyzed. The pH of the feed samples from day 1 to day 55 was determined using a pH meter (Mettler-Toledo, GmbH, Griffin, Switzerland). Organic acids, including lactic, acetic, propionic, and butyric acids, were determined in samples on days 1, 15, and 55 using high performance liquid chromatography (Waters Alliance e2695, Waters, Massachusetts, USA), with reference to the method of Zhao et al. (2020). Carbomix H-NP 10:8% (7.8 mm, 300 mm, 10 mm) was used as the stationary phase at 55°C. The mobile phase was 0.0254% sulfuric acid. The flow rate of the mobile phase was 0.6 mL/min, the sample volume was 10 μL, and the detection wavelength was 214 nm using a UV detector. *Lactobacillus*, *Bacilli*, *Clostridium*, Aerobic bacteria, and *Coliform* bacteria were measured in fecal samples from Bamei piglets on days 2, 20 and 60 using MRS, NA, CLO, NA, and EMB solid medium, respectively, referring to the method of Pang et al. (2011).

### 2.5 Determination of immunity of Bamei piglets

Nine immune-related cytokines were selected as immune indicators to assess the effect of feeding different feeds on the immunity of weaned piglets, including Tumor Necrosis Factor α (TNF-α), Interleukin 2 (IL-2), Interleukin 1β(IL-1β), Interferon γ (INF-γ), Myeloid Differentiation Factor 88 (MyD88), Toll-like receptor 2 (TLR2), Toll-like receptor 4 (TLR4), Interleukin 10 (IL-10), and Nuclear factor-kappa B (NF-KB). These immune-related cytokines in fecal samples on Day 50 were measured using ELISA kits (Beijing Dogesce Biotechnology Co. Ltd., China) with reference to the manufacturer’s instructions and the method of Liu et al. (2022).

### 2.6 Extraction of fecal genomic DNA and sequencing of 16S rDNA

The total bacterial DNA from Bamei piglets’ feces samples on days 10 and 50 was extracted using the Bacterial DNA Kit D3350-02 kit (Omega Biotek, Norcross, GA, USA). DNA quality was assessed by 1% agarose gel electrophoresis and a NanoDrop™2000 spectrophotometer (Thermo Fisher Scientific, Waltham, MA, USA) to assess the quality of the extracted DNA. The 16S rDNA V3-V4 variable region of eligible feces samples was amplified by PCR using primers 338F (5′-ACTCCTACGGGAGGCAGCAG-3′) and 806R (5′-GGACTACHVGGGTWTCTAAT-3′), referring to the method of Wang et al. (2020). After purification and quantification of the PCR products, the purified 16S rDNA amplicons were used to construct a library of PE 2 × 300 using the IlluminaMiSeq platform (Illumina, San Diego, USA). Sequencing was performed using Illumina’s MiSeq PE300 platform (Shanghai Majorbio Bio-pharm Technology Co. Ltd., China).

### 2.7 Sequence analysis

The raw sequenced sequences were quality controlled using Trimmomatic software and then spliced using FLASH software to obtain merge sequences (Magoc and Salzberg, 2011), after which low quality sequences were discarded using QIIME software (Version 1.9.1). The UPARSE software (Version 11)^1^ was used to cluster the sequences based on a 97% sequence similarity to obtain the operational taxonomic units (OTUs), and OTUs tables were generated (Cotta et al., 2004). The RDP classifier was used to annotate each sequence with species classification, and the Silva database was used for sequence comparison of bacteria (Quast et al., 2013; Chen et al., 2015). Alpha diversity indices of bacterial groups were calculated using Mothur software. UPGMA sample clustering trees were constructed using QIIME software. Beta diversity analysis of bacterial populations, RDA analysis, and correlation analysis between bacterial populations and environmental factors were performed using R software,^2^ and the Kruskal-Wallis test was performed for analysis of variance. Linear discriminant analysis Effect Size (LEfSe) analyses were performed using python software (Version 2.7.14).^3^

### 2.8 Statistical analysis

Statistical analysis of the experimental data was carried out using IBM SPSS 22.0 software. One-way ANOVA using Duncan’s multiple range tests was used to calculate significant differences between treatments. *P* < 0.01 and *P* < 0.05 indicate highly significant and significant differences, respectively, while *P* > 0.05 indicates non-significant differences.

## 3 Results

### 3.1 Effect of added strains or antibiotics on feed

**FIGURE 1:**
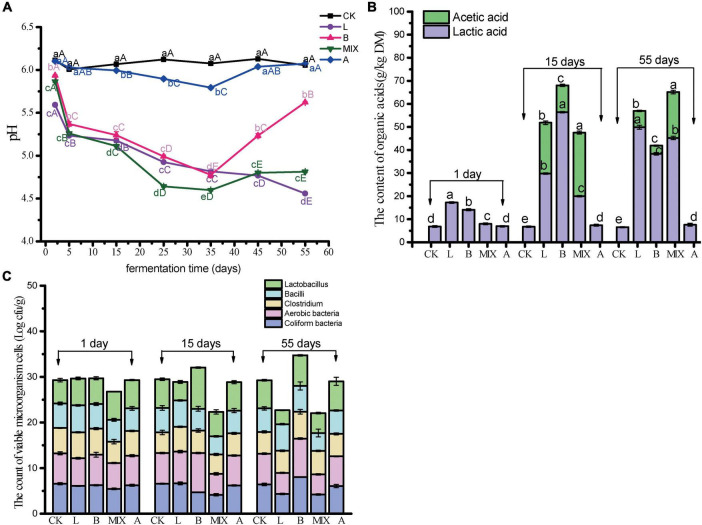
Determination of pH, organic acids and microorganisms in different treatment groups, on different days. **(A)** pH; **(B)** organic acid content at 1, 15 and 55 days, respectively; **(C)** The count of viable microorganism cells at 1, 15 and 55 days, respectively. Different capital letters in the same row indicate significant differences (*P* < 0.05) in values for different periods in the same treatment group, and different lowercase letters in the same column for the same period indicate significant differences (*P* < 0.05) in different treatment groups.

The pH, organic acid content, and microbial counts were recorded from the preparation of the fermented feed to Day 55. The results of the pH measurements for the different treatment groups over the days are shown in Figure 1A. The pH of Group CK did not change significantly over the 55 days and remained at approximately 6.1, while Group A showed a decrease and then an increase. It is worth noting that on day 2 after fermentation, the pH in Group L decreased sharply to 5.59 (*P* < 0.05) and also decreased significantly in Groups B and MIX. pH in Group MIX decreased to the lowest of all observations at 4.59 by day 35 (*P* < 0.05), while Group B showed a significant increase to 5.62 after day 55. The addition of *L. plantarum* QP28-1 to Groups L and MIX maintained the feed in an acidic state for a long period of time, while the addition of *B. subtilis* to Group B reduced and then increased the pH of the feed. This corresponds to the changes in the levels of organic acids detected, as shown in Figure 1B, where low levels of lactic acid were detected in all treatment groups at day 1, with Group L and Group B being significantly higher than the other groups (*P* < 0.05). By day 15, Group B had the highest lactic acid level at 56.38 g/kg DM (*P* < 0.05). It is worth noting that acetic acid was detected in Groups L, B and MIX, with Group MIX having the highest level of acetic acid at 27.56 g/kg DM (*P* < 0.05). By day 55, Group L had the highest level of lactic acid at 49.83 g/kg DM (*P* < 0.05). The results of the microbial counts in the feed are shown in Figure 1C. On days 1 and 15, *Coliform* bacteria, Aerobic bacteria, and *Clostridium* were significantly lower in the MIX group. On day 20, *Lactobacillus* and Aerobic bacteria were higher in Group B than in the other groups. On day 55, *Coliform* bacteria and Aerobic bacteria were higher in Group B than in the other groups, while all bacteria were lower in Groups L and MIX than in the other groups.

### 3.2 Effect of fermented feed on growth performance

The growth performance of the Bamei piglets in different treatment groups was shown in Table 2. The differences in initial weight, final weight, and ADG between the Groups CK, L, B, MIX and A were not significant (*P* > 0.05). The ADFI of Groups B and MIX was significantly higher than that of Group L. The FCR of Group B was the highest, reaching 3.28, while that of Group MIX was significantly lower than that of the other groups (*P* < 0.05).

**TABLE 2:** Comparison of growth performance of Bamei pigs in different treatment groups.

Items	Dietary treatments^3^					SEM^1^	*p*-value^1^
	CK	L	B	MIX	A		
Initial weight (kg)	9.12	8.88	9.04	8.87	8.83	0.114	0.816
Final weight (kg)	29.37	28.65	29.74	31.83	30.06	1.071	0.739
ADG^2^ (kg/day)	0.34	0.32	0.35	0.39	0.36	0.023	0.627
ADFI (kg/day)	1.05^ab^	0.99^b^	1.13^a^	1.14^a^	1.09^ab^	0.018	0.026
FCR	3.10^b^	3.02^bc^	3.28^a^	2.99^c^	3.07^bc^	0.028	0.000

^1^Different lowercase letters “a, b, c” in the same row indicate significant differences (*p* < 0.05). Data represent the mean ± standard deviation of 12 replicates. SEM, standard error of mean.

^2^ADG, average daily gain; ADFI, average daily feed intake; FCR, feed conversion ratio.

^3^Feeding trial lasted 60 days. Group CK was fed a basal diet; Group L was fed *L. plantarum* fermented feed; Group B was fed *B. subtilis* fermented; Group MIX was fed a mixed fermented feed; Group A was fed antibiotic feed.

### 3.3 Analysis of gut microbial community diversity

**FIGURE 2:**
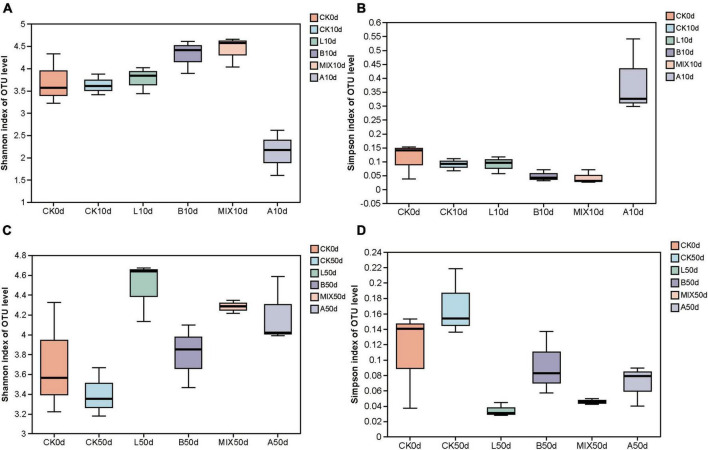
Analysis of the α-diversity of fecal samples from Bamei pigs, including the Shannon and Simpson indexes; **(A)** Shannon index on OUT level at 10 days; **(B)** Simpson index on OUT level at 10 days; **(C)** Shannon index on OUT level at 50 days; **(D)** Simpson index on OUT level at 50 days.

High-throughput sequencing was used to analyze the structure of the bacterial microbiota in the feces of the different treatment groups at 0, 10, and 50 days, respectively, for each sample. Bacterial community alpha diversity was expressed using the Shannon and Simpson indices. The results, presented in Figure 2, showed that at day 10, the Shannon index was slightly higher and the Simpson index was lower in Groups L, B and MIX compared to pre-fermentation, while the Shannon index was lower and the Simpson index was higher in Group A. By day 50, the Shannon index was highest in Group L, and higher in Groups B, MIX, and A than in Group CK. The Shannon and Simpson indices were not significantly different between the treatment groups.

**FIGURE 3:**
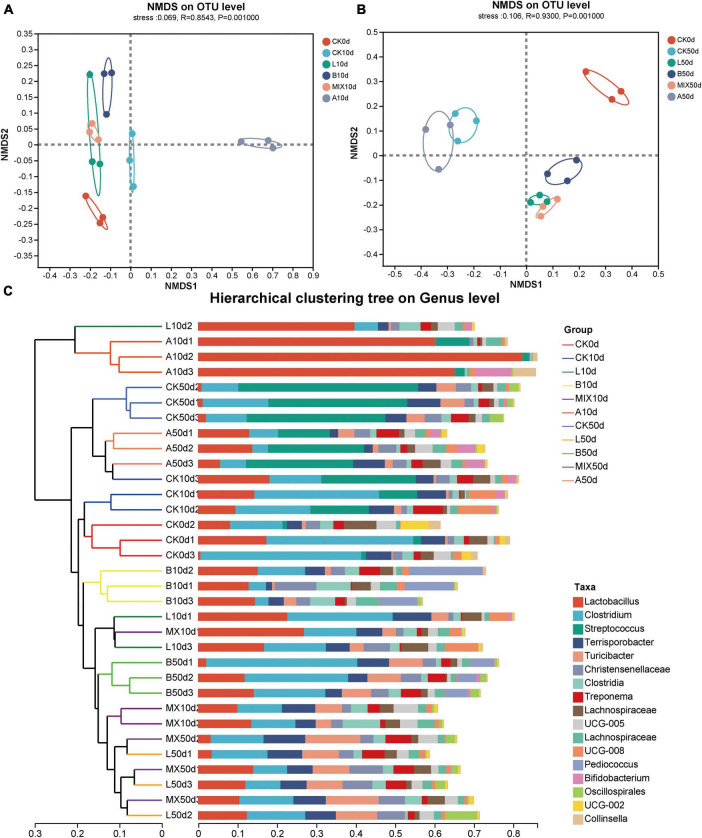
Analysis of β-diversity of fecal samples from Bamei pigs. Non-metric multidimensional scaling analysis (NMDS) analysis of bacterial community compositions at 10 days **(A)** and 50 days **(B)**; **(C)** sample clustering analysis on genus level.

NMDS analysis (non-metric multidimensional scaling analysis) was used to analyze the β-diversity of the community structure of Bamei piglets in different treatment groups at 10 and 50 days. As shown in Figure 3, there was a clear separation of bacterial clusters between the treatment groups, indicating that the addition of either *L. plantarum* QP28-1a or *B. subtilis* QB8a or antibiotics significantly altered the bacterial community structure of the intestinal microbiota. On day 10, Figure 3A, the separation between the treatment groups became more pronounced, with Groups L, B and MIX with the addition of exogenous strains, being closer in structure to the bacterial community than the Groups CK and A. At day 50, Figure 3B, the bacterial microbiota of each treatment group was still separated from the other treatment groups, especially in Group A.

In order to show the similarities and differences between the bacterial community structures of the different treatment groups in more detail, a cluster analysis was performed on the sample community distance matrix, and a sample hierarchical clustering tree was constructed, as shown in Figure 3C. The majority of the samples from the treatment groups were clustered together, which was consistent with the results in Figures 3A, B.

### 3.4 Comparison of gut bacterial community composition

**FIGURE 4:**
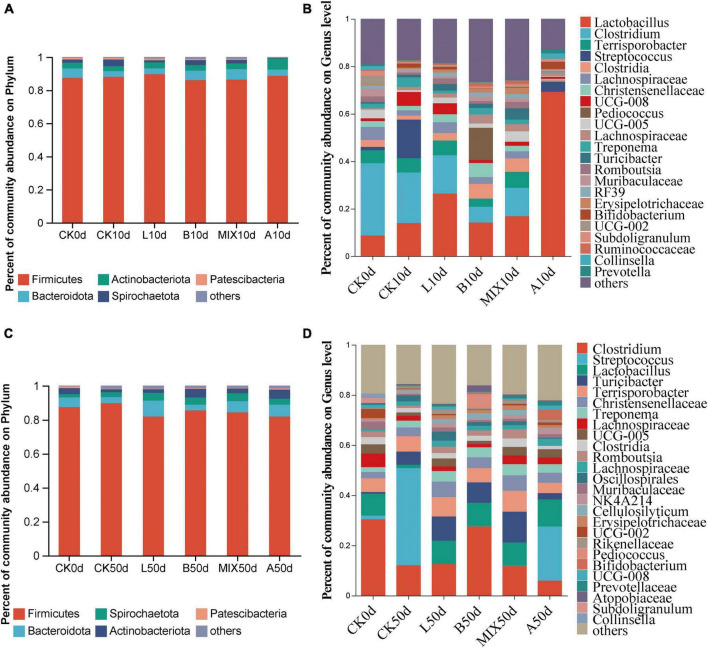
Community bar plots analysis and bacterial relative abundances analysis by **(A)** phylum at 10 days; **(B)** genus at the 10 days; **(C)** phylum at the 50 days; **(D)** genus at the 50 days.

The relative analysis of bacteria in the feces of Bamei piglets at days 10 and 50 was carried out. At the phylum level, Firmicutes was the dominant phylum at days 10 and 50, followed by Bacteroidota. At day 10, Figure 4A, the relative abundance of Bacteroidota was increased in the B and MIX groups compared to Group CK, and at day 50, as shown in Figure 4C, the Group L reduced the relative abundance of Firmicutes (81.8%) compared to Group CK (89.9%) and increased the relative abundance of Bacteroidota (9.4%) compared to Group CK (3.6%). At the genus level, Figure 4B, *Lactobacillus* 8.7%, *Clostridium* 30.5%, and *Terrisporobacter* 5.5% dominated the competition prior to feed fermentation. At day 10, the relative abundance of *Lactobacillus* increased and the relative abundance of *Clostridium* and *Streptococcus* decreased significantly in Groups L and MIX. *Lactobacillus* was increased in Group B. It is worth noting that the relative abundance of *Lactobacillus* in Group A was significantly higher than the other treatment groups at day 10, reaching 69%, with *Lactobacillus* and *Streptococcus* taking a competitive advantage. By day 50 in Figure 4D, the relative abundance of *Lactobacillus* in Groups L, B and MIX A was 9.3, 9.4, 9.2, and 10.8%, respectively, compared to 1.3% in Group CK. The relative abundance of *Streptococcus* in Groups L, B and MIX decreased sharply compared to Group CK.

**FIGURE 5:**
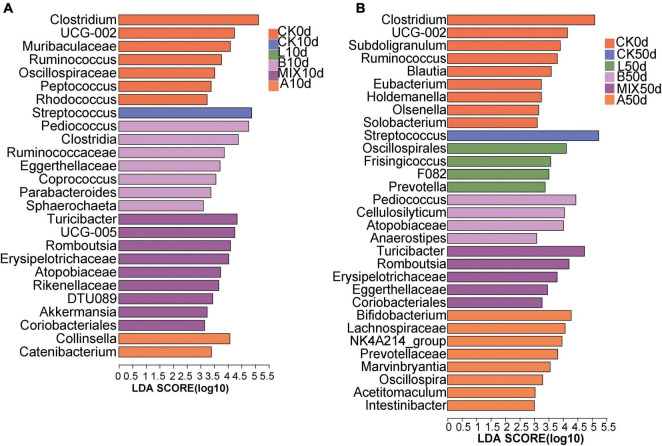
Comparison of microbial variations using LEfSe analysis for 10 days **(A)** and 50 days **(B)**. *P* < 0.05, LDA Score > 3.0 on genus level.

Differences in the structure of bacterial microbiota composition between treatment groups were further explored by the method of Lefse. At the genus level, the relative abundance of *Clostridium*, *Muribaculaceae*, *Ruminococcus*, and *Oscillospiraceae* was significantly higher in the CK0d group than in the other treatment groups. At 10 days in Figure 5A, Group CK was significantly enriched in *Streptococcus*, and the Group B was significantly enriched in the genera *Pediococcus*, *Clostridia*, and others. *Turicibacter*, *Romboutsia*, and *Erysipelotrichaceae* were dominant in Group MIX, *Collinsella*, *Catenibacterium* predominated in Group A. By the day 50, Figure 5B, the Group L was significantly enriched in the genera *Oscillospirales*, *Frisingicoccus*, and *Prevotella*. The significantly different genera in Group B were *Pediococcus*, *Cellulosilyticum*. The significantly different genera in Group MIX included *Turicibacter*, *Romboutsia*, and the significantly enriched genera in Group A included *Bifidobacterium*, *Lachnospiraceae*, and *Prevotellaceae*.

### 3.5 Relationship between microbial community structure and immune cytokines

**FIGURE 6:**
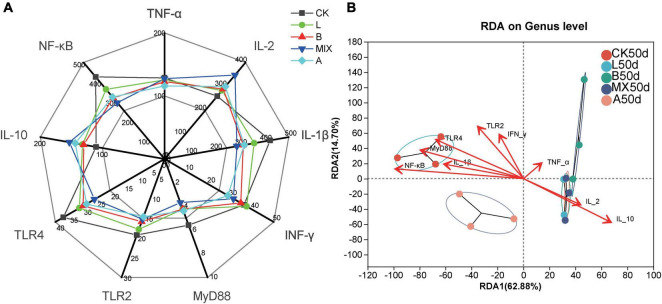
Analysis of the effect of feeding different diets for 50 days on the immunity of Bamei pigs. **(A)** Measurement of immune-related cytokine expression after 50 days of feeding different feed, including Tumor Necrosis Factor α (TNF-α, pg/mL), Interleukin 2 (IL-2, pg/mL), Interleukin 1β (IL-1β, pg/mL), Interferon γ (INF-γ, pg/mL), Myeloid Differentiation Factor 88 (MyD88, ng/mL), Toll-like receptor 2 (TLR2, ng/mL), Toll-like receptor 4 (TLR4, ng/mL), Interleukin 10 (IL-10, pg/mL) and Nuclear factor-kappa B (NF-KB, ng/mL). **(B)** Redundancy analysis (RDA) analysis show the correlations between the genus bacterial community structures and immune-related cytokines using the 16S rRNA gene sequences of fecal samples from Bamei pigs at Day 50.

ELISA kits were used to detect changes in the levels of several signature immune cytokines, including TNF-α, IL-2, IL-1β, INF-γ, MyD88, TLR2, TLR4, IL-10, and NF-KB, in fecal samples from Bamei piglets to analyze the effect of fermented feed on immunity. The results, presented in Figure 6A, showed that, compared with Group CK, the Groups L, B, MIX, and A not only down-regulated the expression of the pro-inflammatory factors TNF α and IL-1β, but also up-regulated the expression of the anti-inflammatory factors IL-2 and IL-10, improving the immunity of the Bamei piglets, with the Group MIX having the most significant effect (*P* < 0.05). Meanwhile, the expression of immune factors TLR2, TLR4, MyD88, and NF-KB was down-regulated in Groups L, B, MIX and A compared to Group CK.

To visualize the relationship between feces sample distribution and immune factors, RDA analysis was used, Figure 6B. The results showed that the eigenvalues of axis 1 and 2 were 0.6288 and 0.1470, respectively. The RDA results revealed correlations between the bacterial genus level community composition of samples from different treatment groups with different immune cytokines. Samples in the same treatment group clustered together, while there was a clear separation between the different treatment groups. The distribution of the bacterial community structure in the treatment groups L50d and MX50d showed a strong positive correlation with the immune factors IL-2 and IL-10, the distribution of CK50d showed a strong positive correlation with NF-KB, MyD88, TLR4, IL-1β, and A50d and B50d showed a lower selectivity with the immune cytokines. The results of the RDA analysis showed that the expression of the immune cytokines IL-2 and IL-10, NF-KB, MyD88, TLR4, and IL-1β was significantly correlated with the changes in bacterial community in the fecal samples of the tested Bamei piglets.

**FIGURE 7:**
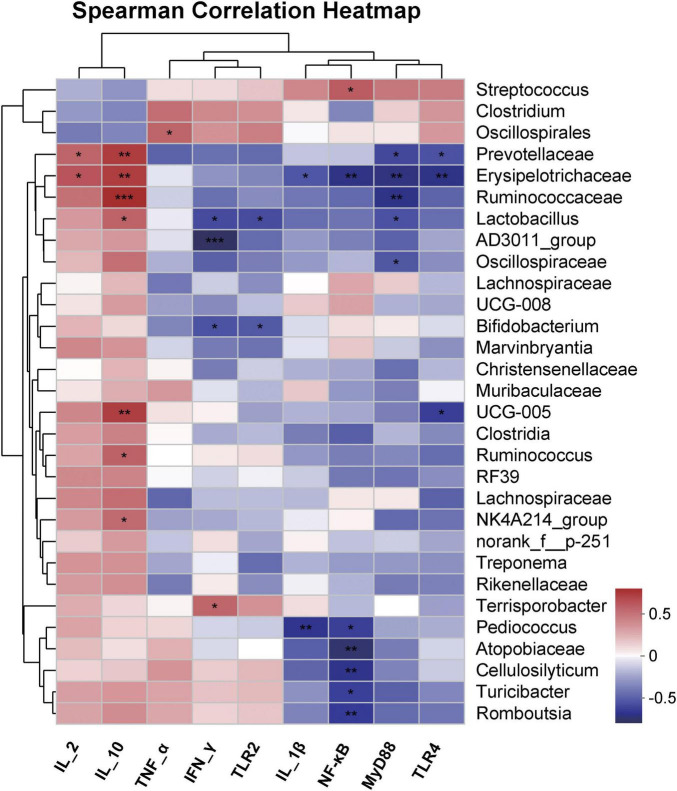
Spearman correlation heatmap of abundance of the top 30th abundant bacterial genus and immune-related cytokines fecal samples from Bamei pigs at 50 days. *P < 0.05; **P < 0.01; ***P < 0.001.

The correlation between the bacterial community and immune cytokines was further elucidated by Spearman’s correlation analysis after 50 days of feeding fermented feed to Bamei piglets (Figure 7). The relative abundance of *Lactobacillus* was positively correlated with IL-2, significantly correlated with IL-10 (*P* < 0.05), and negatively correlated with INF-γ, TLR2, and MyD88 after 50 days of fermented feed. The relative abundance of *Erysipelotrichaceae* showed a significant positive correlation with IL-2 (*P* < 0.05), a highly significant positive correlation with IL-10 (*P* < 0.01), a significant negative correlation with IL-1β (*P* < 0.05), a highly significant negative correlation with TLR4, MyD88, and NF-κB (*P* < 0.01). The relative abundance of *Ruminococcaceae* showed a highly significant positive correlation with IL-10 (*P* < 0.01) and a highly significant negative correlation with MyD88 (*P* < 0.01). The relative abundance of *Streptococcus* was significantly positively correlated with NF-κB (*P* < 0.01). *Bifidobacterium* was significantly negatively correlated with INF-γ, TLR2 (*P* < 0.05). The relative abundance of *Prevotellaceae* showed a significant positive correlation with IL-2 (*P* < 0.05), a highly significant positive correlation with IL-10 (*P* < 0.01), and a significant negative correlation with TLR4 and MyD88 (*P* < 0.05).

## 4 Discussion

In recent years, the restricted use of antibiotics in farming has driven the development of research on the screening and application of green antimicrobials, and probiotics such as *Lactobacillus* and *Bacillus* have been applied in pig farming. In this study, the hypothesis that probiotic fermented feed can improve the intestinal microorganism environment, promote growth, and improve the immunity of Bamei piglet was verified in four main dimensions: feed, growth performance, gut microbiota, and immunity.

### 4.1 Effect of adding probiotics to feed

In the fermented feed, the pH of Groups L, B, and MIX was significantly reduced by the addition of *L. plantarum* or *B. subtilis* (Figure 1A) and the fermentation process was accompanied by the production of lactic acid and acetic acid (Figure 1B). Previous studies have shown higher lactic acid concentrations and lower pH were important for the antibacterial properties of fermented feed, which also work with the intestinal mucosa to form a bacterial film barrier that can inhibit the colonization and proliferation of pathogenic bacteria (van Winsen et al., 2001a; Klewicki and Klewicka, 2004), which is also in line with the results of our analysis of fermented feeds for harmful bacterial (Figure 1C). Similarly, Tajima et al. (2010) used *L. plantarum* to ferment liquid feed, and the pH of the feed was reduced to 3.93, the lactic acid content reached 236.8 mmol/g after fermentation. Feeding of this feed by animals increased the acidity of the gastrointestinal tract, which helped to enhance the activity of digestive enzymes and improve digestive capacity (Tajima et al., 2010). In summary, *L. plantarum* and *B. subtilis* mainly produce probiotic substances, organic acids, and digestive enzymes through their own metabolism to inhibit the growth of harmful bacteria and promote nutrient absorption to fulfill their probiotic roles.

### 4.2 Effect of fermented feeds on growth and feed intake of weaned piglets

*L. plantarum* and *B. subtilis* may produce active digestive enzymes during the fermentation of feed, which increase the palatability and nutrition of feeds, and contribute to the improvement of animal growth performance (Santoso et al., 1995; Wang et al., 2014; Le et al., 2016; Dai et al., 2017). There were many reports on the effects of fermented feed on piglet growth performance, but the results were not identical. Jatkauskas and Vrotniakiene (2009) found higher feed intake, average daily gain, and feed conversion in the test group than in the control group after feeding *Enterococci* to calves. Canibe and Jensen (2003) showed that the growth rate and feed intake of weaned piglets were significantly improved by feeding piglets with lactic acid bacteria liquid fermented feed. Our results showed that there was no significant change in ADG between the treatment groups, however, the ADFI was significantly lower in Group L compared to Group CK and the FCR was significantly lower in Group MIX compared to Group CK. The reason for the reduced FCR may be that mixed fermentation can increase the beneficial metabolites in the feed and improve the nutritional level of the feed. It has been suggested that the growth promoting effect of fermented feed is closely related to the strain of fermented feed, the fermentation process, the amount of additives, the nutritional composition of the feed, and the growth stage of the pigs, thus leading to different results from one study to another (van Winsen et al., 2001b; Wang et al., 2014).

### 4.3 Fermented feed improves intestinal microbiota in Bamei piglets

The contribution of fermented feed to the probiotic properties of the intestinal microbiota was mainly reflected in the modification of the diversity, structure and abundance of the microbiota and the maintenance of intestinal homeostasis. On the one hand, probiotics can increase the diversity of the intestinal microbiota and inhibit the growth and reproduction of pathogens, thus achieving the maintenance of a dynamic balance in the intestinal microbial community (Kale-Pradhan et al., 2010). Similarly, Tajima et al. (2010) found that liquid fermented feeds with LAB significantly increased the Shannon and Chao1 indices of feces microbiota in weaned piglets. In contrast, α-diversity was reduced in Group A with antibiotics. On the other hand, feeding probiotic fermented feed improved the structure of the intestinal microbiota, resulting in a reduction in pathogens and an increase in beneficial bacteria in the pig intestine. The fermentation process of LAB will produce a variety of organic acids, mainly lactic acid, so that the pH value of the feed is reduced, which destroys the neutral, aerobic environment for the growth of some harmful bacteria. After ingestion by animals, acid-resistant bacteria such as *Lactobacillus* enter the intestinal tract and form a dominant species. Demeckova et al. (2002) showed a significant reduction (*P* < 0.01) in *E. coli* in the feces of sows fed a liquid fermented feed with *L. plantarum*. Similarly, van Winsen et al. (2002) found that *L. plantarum* liquid fermented feed significantly increased the number of LAB in the feces of pigs while significantly reducing the number of *Salmonella* and *E. coli*. After 10 days of feeding the fermented feed (Figure 4B), the relative abundance of probiotic *Lactobacillus* in Group L and Group A increased significantly, especially in the antibiotic group, indicating that feeding *L. plantarum* fermented feed and the antibiotic feed benefited the growth of *Lactobacillus* in the intestine. Notably, the Groups L, B, and MIX all showed a near disappearance of *Streptococcus*, an opportunistic pathogen such as Group B *Streptococcus* present in the intestinal and genitourinary tracts (Madrid et al., 2017), as well as caries-causing *Streptococcus mutans* (DeWitte and Bekvalac, 2010), suggesting that the fermented feed effectively reduced the risk of these diseases. By day 50, *Clostridium*, *Lactobacillus*, *Terrisporobacter* and were still the dominant genus, and *Streptococcus* in Groups L, B and MIX were still suppressed. More details are revealed through LEFSe analysis. The Group CK was significantly enriched in opportunistic pathogens such as *Streptococcus* and *Clostridium*. In Group L, the probiotic *Oscillospirales* was significantly enriched at day 50 (log10 LDA ≥ 3.0), a slightly enigmatic but probiotic associated with intestinal health (Konikoff and Gophna, 2016). In short, the fermented feed did improve the intestinal microbiota of Bamei piglets.

### 4.4 Fermented feed improves immunity in weaned Bamei piglets

Cytokines are biologically active protein-peptide molecules synthesized mainly by immunologically active cells, and have the function of mediating and regulating the immune response (Farthing, 2004; Donkor et al., 2012). Studies have found that probiotic fermented feed has a wide range of effects, both in promoting or regulating the balance of the organismal microbiota, promoting nutrient absorption, resisting invasion by foreign microorganisms, and improving immunity, especially intestinal immunity (Markowiak and Slizewska, 2018). Probiotics regulate immunity mainly by enhancing the viability of lymphocytes and B cells, inducing T cell differentiation, and producing cytokines, thus releasing and regulating the expression of immune-related cytokines, such as IL-2, IL-10, TNF-α, IL-1β, and IFN-γ (Farthing, 2004; Donkor et al., 2012; Infusino et al., 2020; Mazziotta et al., 2023). Previous studies have found that probiotic fermented feed improves the immunity of animals in three main ways, including pro-inflammatory cytokines, anti-inflammatory cytokines and typical immune-related signal pathways. On the one hand, pro-inflammatory cytokines IL-1β and TNF-α expression levels were upregulated when the organism was infected by pathogenic bacteria, producing a mucosal inflammatory response (Papadakis and Targan, 2000). On the other hand, some probiotics can induce the differentiation of Th2 cells and cause the expression of anti-inflammatory cytokines IL-2 and IL-10, reducing the damage caused by the inflammatory response (Lopez et al., 2011). Ling et al. (2015) found that *Clostridium butyricum* combined with the *Bifidobacterium infantis* probiotic mixture down-regulated IL-1β and TNF-α and up-regulated IFN-γ, IL-10 in the fractional intestine and alleviated inflammation. Sanchez-Munoz et al. (2008) reported that the addition of LAB to the feed reduced the concentrations of the pro-inflammatory cytokines TNF-α and IL-1β and produced an anti-inflammatory effect on the intestine of fish, which is consistent with our results. The results of our study showed that both Group L, B and MIX with the addition of *L. plantarum* QP28-1a or *B. subtilis* QB8a, and the antibiotic group had lower pro-inflammatory cytokines IL-1β and TNF-α and higher anti-inflammatory cytokines IL-2, and IL-10, thus the immunity was significantly improved, especially Group MIX, which had the most significant immune enhancement effect, even better than the antibiotic group (Figure 6A). In addition to some of the immune-related cytokines mentioned above, signal pathways, especially the TLR4/MyD88/NF-κB signal pathway, play an important role in the regulation of inflammation and immunity in the body (Devi et al., 2015; Ciesielska et al., 2021). A study found that weaning activates intestinal inflammatory signaling pathways, such as MAPK and NF-kB (Hu et al., 2013). When bacteria invade the intestinal mucosa of weaned piglets, they activate the TLR4/MyD88/NF-KB inflammatory signaling pathway, promoting inflammatory factor transcription and triggering inflammation (Buer and Balling, 2003). Li et al. (2016) also found that *Lactobacillus acidophilus* could alleviate *E. coli*-induced intestinal inflammation by inhibiting the signal pathways of TRLs and NF-KB. Similarly, our results showed that the expression of cytokines TRL2, TRL4, MyD88, and NF-KB in the pig intestine was significantly reduced after 50 days of feeding fermented diets supplemented with *L. plantarum* QP28-1a or *B. subtilis* QB8a compared to Group CK, implying that the fermented feed reduced intestinal inflammation, and that Group MIX was even more effective than the antibiotic group (Figure 6A). The results of the RDA analysis showed that a significant separation occurred between the different treatment groups due to different intestinal microbiota structures. Notably, the 16S rRNA gene sequences of Groups L, B and MIX supplemented with *L. plantarum* QP28-1a or *B. subtilis* QB8a were significantly positively correlated with the anti-inflammatory cytokines IL-2 and IL-10 and negatively correlated with IL-1β, TRL4, MyD88, and NF-κB. In contrast, IL-1β, TRL4, MyD88, and NF-KB were significantly correlated with bacterial communities in Group CK. This again provides strong evidence that Groups L, B and MIX supplemented with *L. plantarum* QP28-1a or *B. subtilis* QB8a upregulated anti-inflammatory cytokines, downregulated pro-inflammatory cytokines, and inhibited the TRL4/MyD88/NF-KB signal pathway, thereby improving immunity. The relationship between specific bacterial genera and cytokines showed that *Lactobacillus* positively correlated with the anti-inflammatory cytokine IL-2 and significantly correlated with IL-10 (*P* < 0.05), and also significantly inhibited the expression of the pro-inflammatory cytokines TRL2 and MyD88. *Streptococcus* was significantly positively correlated with NF-KB, while few *Streptococcus* were found in Groups L, B and MIX (Figure 4). In addition, *Prevotellaceae*, *Erysipelotrichaceae*, and *Ruminococcaceae* were also all significantly negatively correlated with pro-inflammatory cytokines and significantly positively correlated with anti-inflammatory cytokines. In summary, the fermented feed with *L. plantarum* QP28-1a or *B. subtilis* QB8a significantly improved the immunity of pigs by upregulating anti-inflammatory cytokines, downregulating pro-inflammatory cytokines, and inhibiting the TRL4/MyD88/NF-KB inflammation-related signaling pathway.

## 5 Conclusion

Our study found that fermentation of feed by either *L. plantarum* QP28-1a or *B. subtilis* QB8a lowered the pH (*P* < 0.05) of the feed and was accompanied by the production of lactic acid and acetic acid, resulting in a reduction of harmful *Coliform* and *Clostridium*, extending the shelf life, and improving the quality of the feeds. The FCR was significantly lower in Group MIX which was fed a mixed fermented diet for 60 days (*P* < 0.05). High-throughput sequencing results showed that the fermented feed increased the α-diversity of the intestinal microbiota and significantly altered the β-diversity, increasing the relative abundance of beneficial bacteria such as *Lactobacillus* and *Turicibacter* and decreasing the relative abundance of opportunistic pathogens such as *Streptococcus* and *Clostridium*, contributing to the maintenance of intestinal health. On the other hand, the fermented feed also had a significant and positive effect on the immunity of Bamei piglets, not only significantly up-regulating the expression of anti-inflammatory cytokines IL-2 and IL-10, but also significantly down-regulating the expression of pro-inflammatory cytokines TNF-α and IL-1β, while inhibiting the TLR4/MyD88/NF-kB signaling pathway to alleviate intestinal inflammation. The results presented demonstrated the excellent application potential of *L. plantarum* QP28-1a in combination with *B. subtilis* QB8a for fermented feed, and that this method of feeding fermented feed to weaned piglets was worth promoting.

## Data Availability

The datasets presented in this study can be found in online repositories. The names of the repository/repositories and accession number(s) can be found below: http://www.ncbi.nlm.nih.gov, with accession numbers OM049403 and OQ703036.

## Footnotes

1 http://www.drive5.com/uparse/ (accessed April 2, 2023)

2 http://www.r-project.org (accessed February 11, 2023)

3 https://www.python.org/ (accessed February 17, 2023)
